# Supplementary material for: Analysis of PPARγ Signaling Activity in Psoriasis
Source: Int J Mol Sci. 2021 Aug 10;22(16):8603. doi: 10.3390/ijms22168603 (PMC8395241; doi:10.3390/ijms22168603)
Supplement: Supplementary file 1 [file ijms-22-08603-s001.zip › Supplemental materials_Analysis of PPARg signaling activity in psoriasis/Pathway models/Models images and html files/PPARG inhibits phenotypes assotiated with psoriasis/280626.html]

skin damage


# Disease skin damage

|  |  |
| --- | --- |
| URN | urn:agi-disease:skin%20damage |
| Total Entities | 0 |
| Connectivity | 3350 |
| Name | skin damage |

---

|  |  |
| --- | --- |
| Pathway | Argininosuccinic Aciduria in Urea Cycle Disorders |
|  | PPARG inhibits phenotypes assotiated with psoriasis |
|  | efects selected |

---

|  |  |
| --- | --- |
| MedScan ID | 9104487 |

---

|  |  |
| --- | --- |
| Alias | epithelial defects |
|  | skin loss |
|  | epidermal lesions |
|  | cutaneous lesions |
|  | defect of the skin |
|  | skin defect |
|  | lesion in the skin |
|  | skin losses |
|  | breakdown of skin |
|  | lesions of the skin |
|  | damages of the skin |
|  | skin damages |
|  | skin damage |
|  | skin breakdown |
|  | skin defects |
|  | cutaneous lesion |
|  | losses from the skin |
|  | lesions on the skin |
|  | skin lesions |
|  | cutaneous defects |
|  | breakdowns of skin |
|  | dermoid lesions |
|  | skin ruptures |
|  | epidermis lesions |
|  | skin lesion |
|  | epithelial defect |
|  | skin rupture |
|  | lesions of the epidermis |
|  | lesion on the skin |
|  | skin breakdowns |
|  | dermis lesions |
|  | cutaneous defect |
|  | dermal lesions |
|  | lesions in the dermis |
|  | defects of the skin |
|  | damage of skin |

---
